# A Comparison of Human and Computational Melody Prediction Through Familiarity and Expertise

**DOI:** 10.3389/fpsyg.2020.557398

**Published:** 2020-12-09

**Authors:** Matevž Pesek, Špela Medvešek, Anja Podlesek, Marko Tkalčič, Matija Marolt

**Affiliations:** ^1^Faculty of Computer and Information Science, University of Ljubljana, Ljubljana, Slovenia; ^2^Faculty of Arts, University of Ljubljana, Ljubljana, Slovenia; ^3^Faculty of Mathematics, Natural Sciences and Information Technologies, University of Primorska, Koper, Slovenia

**Keywords:** music similarity, music perception, music information retrieval, implication-realization model, compositional hierarchical model, melody prediction

## Abstract

Melody prediction is an important aspect of music listening. The success of prediction, i.e., whether the next note played in a song is the same as the one predicted by the listener, depends on various factors. In the paper, we present two studies, where we assess how music familiarity and music expertise influence melody prediction in human listeners, and, expressed in appropriate data/algorithmic ways, computational models. To gather data on human listeners, we designed a melody prediction user study, where familiarity was controlled by two different music collections, while expertise was assessed by adapting the Music Sophistication Index instrument to Slovenian language. In the second study, we evaluated the melody prediction accuracy of computational melody prediction models. We evaluated two models, the SymCHM and the Implication-Realization model, which differ substantially in how they approach melody prediction. Our results show that both music familiarity and expertise affect the prediction accuracy of human listeners, as well as of computational models.

## 1. Introduction

One of the main aspects of listening to music is the tendency of the brain to constantly predict the upcoming melodic events. How human listeners perform the ongoing prediction of music is influenced by (i) their general music expertise and by (ii) their familiarity with the type of music they are listening to. These two concepts are two facets of the knowledge that listeners possess. Music knowledge, in the widest sense, is acquired in various ways, either through formal music training or just by listening to music regularly. The research topic of melody prediction has been extensively studied from the perspective of psychology (e.g., Rohrmeier and Koelsch, 2012; Egermann et al., 2013) and neuroscience (e.g., Zatorre et al., 1994; Thiessen and Saffran, 2009; Lappe et al., 2013). In recent years, research on understanding human melody prediction has crossed over to the development of computational models that perform melody prediction. The knowledge that such models use, typically stems from a dataset that the researchers develop or train their models on. The question is how do models, trained on a dataset, and humans, trained through years of listening to music, compare in terms of melody prediction. We conjecture that the algorithms, just like humans, perform better at melody prediction on familiar music, i.e., on music that resembles the music they have been trained on. We also conjecture that algorithms that are agnostic of music culture perform equally well on music from different cultures. We compare melody prediction performance of the algorithms to that of humans and shed light on how much expertise and familiarity is required to train an algorithm to perform comparably well to humans.

### 1.1. Problem Formulation

The problem of melody prediction can be posed and evaluated in two ways. In a strict way, the predictor (human or algorithm) needs to predict the exact note that follows an initial set of notes of a song. In this case, there is only one possible correct prediction: i.e., the next note in that particular song. However, we can relax the requirements for prediction and treat as correct each prediction that makes sense from a musical perspective. For example, the songs *If You Don't Know Me By Now* by Harold Melvin and the Bluenotes and *Take it to the Limit* by The Eagles start with an identical sequence of notes but at some point begin to diverge^1^. If a predictor was asked to predict the first note that diverges, the correctness of the prediction would depend on whether the ground truth was the first or the second song. In this work we use both strict and relaxed evaluation—for the latter, we devise a **methodology for evaluating melody prediction based on music theory**.

Before proceeding to the next research question, we need to define the terms familiarity and expertise. They are both related to knowing music, but in ways that make them two separate concepts. The term expertise is related either to a more formal way of obtaining broad musical knowledge or intense engagement with music, whereas familiarity is related to the informal acquisition of musical knowledge. One could argue that being an expert in music makes one also familiar with (a specific type of) music. However, with different music cultures, we may encounter different expertise-familiarity scenarios. If we consider European and Chinese listeners and Chinese music, four scenarios are possible: (i) low general music expertise and low familiarity with Chinese music (e.g., a European listener with low music education), (ii) low general music expertise and high familiarity with Chinese music (e.g., a Chinese listener with low music education), (iii) high general music expertise and low familiarity with Chinese music (e.g., a European listener with high musical knowledge), and (iv) high general music expertise and high familiarity with Chinese music (e.g., a Chinese listener with high music education). While the cases (i) and (iv) are intuitive, (ii) and (iii) require more attention. In the case of (ii), the listener has not received formal music education but has been exposed to Chinese music throughout her life. The assumption is that this exposure has given the listener implicit music knowledge. This knowledge could allow her to continue correctly a melody, if it is similar to the melodic patterns (i.e., sequences of notes with similar characteristics) that she has been exposed to although she might not be able to describe why. In the case of (iii), the listener has high general musical knowledge but has not been exposed to Chinese music. In the melody continuation scenario, the listener could likely choose a tone that is correct from the musical theory perspective, but happens rarely in Chinese music. Hence, expertise and familiarity, although correlated, should be treated as two separate variables.

The examples in the previous paragraph are illustrative only and do not reflect our experimental design. In section 3, we describe in detail which familiarity categories (with European or Chinese music) and which expertise categories the subjects fall into. Furthermore, we must distinguish between the concepts of expertise and familiarity on one hand and how we operationalize them for the purpose of our study on the other hand. As described in section 3, we operationalize the expertise using the Musical Sophistication Index instrument (Müllensiefen et al., 2014). In order to operationalize the familiarity we used the cultural background, e.g., we assume Europeans are not familiar with Chinese music but are familiar with European music.

In addition to the question about the melody prediction, we also address the following question: **Do expertise and familiarity influence the melody prediction of humans?** In order to answer this question we devised an experiment where we asked subjects from a homogeneous cultural background (Slovenians with different levels of musical expertise) to predict the melody of Western (i.e., familiar) and non-Western (Chinese, i.e., non-familiar) songs. In order to measure their expertise, we **adapted the Music Sophistication Index (MSI) instrument (Müllensiefen et al.**, **2014****) to the Slovenian language**, which is an additional contribution of this paper.

Finally, we evaluate **how algorithms perform in melody prediction**. We evaluated the performance of two algorithms, (i) the Implication-Realization (I-R) model developed by Narmour (1990), which is agnostic of musical culture, and (ii) the Compositional Hierarchical Model for symbolic music representations (SymCHM) developed by Pesek et al. (2017b), which is trained on a dataset of songs and hence biased toward familiar songs.

## 2. Related Work

In this section, we first survey work on human music prediction from psychology and neuroscience, which demonstrates that familiarity and expertise influence melody prediction, supporting our rationale for the work presented. We then proceed on surveying algorithms for melody prediction, in particular how they use existing knowledge and what methodologies have been proposed for melody prediction. Lastly, we survey work on tonal hierarchies that will be used as the theoretical background for constructing the relaxed evaluation criterion.

### 2.1. Culturally-Dependent Human Melody Prediction

Music from different cultures activates similar brain areas, as shown by Morrison and others in their functional magnetic resonance imaging experiment (Morrison et al., 2003), but there are differences in brain activity between the situations in which one listens to the music of their own cultural background vs. that of a foreign culture. The cultural influence has also been demonstrated for genre preference (Soley and Hannon, 2010), perceived mood estimation (Balkwill and Thompson, 1999), and musical memory (Demorest et al., 2008), among other aspects. Children already show preferences for music similar to their cultural and musical tradition (Demorest et al., 2008; Soley and Hannon, 2010). The preference also affects musical memory, which is better for culturally similar music in comparison to unknown music (Demorest et al., 2008). Also the recognition of rhythm is influenced by culture, which is why foreign rhythms seem more complex and are more difficult to recognize (Cross, 2001).

The cognitive processes which occur in music perception are also employed in language perception, which is supported by the research performed by Nan et al. (2006) and Maess et al. (2001). In the latter, magnetoencephalography was used to analyze the Broca's area in the human brain, which plays an important role in syntactic analysis during auditory language comprehension. The authors showed it also analyzes the incoming harmonic sequences. Nan et al. (2006) further explored the shared functionality of this area and analyzed the structure of a musical phrase. They discovered that the brain response produced by musical phrase inconsistencies are similar to those produced when observing syntactic inconsistencies. Specifically, the *closure positive shift* (CPS), which is a universal phrase perception mechanism and occurs in both music and language domains, was observed. In music, the CPS occurred between 100 and 450 ms after the phrase end. The CPS occurred earlier when the participants listened to music of their culture and later when listening to music of a foreign culture (Nan et al., 2006).

Mood and emotion recognition in music depend on culture-specific and universal structural characteristics of the music played. Balkwill and Thompson (1999) explored whether Western listeners recognize the *intended* emotions in music in the Hindi tonal system, which was unknown to the listeners. The results showed that the listeners are able to recognize basic emotions, e.g., joy, sadness, and anger, however, they are unable to identify more complex emotions which may be different from culture to culture, e.g., “peace” (Balkwill and Thompson, 1999). Fritz et al. (2009) also carried out a survey in the reverse direction and reached a similar conclusion: in Western music, the expression of basic emotions is universally recognizable, as well.

Several studies analyze the cultural influence on rhythmic and melodic complexity (Eerola et al., 2006; de Fleurian et al., 2017). Groups of participants with different cultural backgrounds (African, Western) perceived music of their own culture as less complex. Interestingly, African participants perceived Western music as less complex, compared to the perception of African music by European participants. The perception of Western music as less complex is most likely the consequence of the prevalence of Western culture. People are acquainted with Western music, regardless of their culture. This influence of Western music also results in the disappearance of less robust cultures or, in less radical cases, the infiltration of Western musical foundations into other musical cultures (Huron, 2008).

The topic of musical expectation is also important in both perceptual and cultural contexts. One of the biggest factors accrediting to the differences in music expectations is the culture (Castellano et al., 1984; Krumhansl, 1999; Krumhansl et al., 2000). The studies performed by Castellano et al. (1984) and Krumhansl (1999); Krumhansl et al. (2000) took into consideration two groups of participants—one from a Western culture (European or American) and one from a non-Western culture (African or Asian). The task in these experiments was to predict the continuation of musical excerpts (i.e., a subsequence of notes from a longer sequence). Most commonly, the participants from different cultures have decided very similarly with regard to the continuation of their own and foreign cultures. In one of the studies by Krumhansl (1999), the non-European participants achieved higher accuracy for European music, for the previously known European classical works. Consequently, their responses were more in line with the music theory, compared to the European group, who supposedly possessed more general knowledge of Western music.

Listeners also provide diverse responses to music structure of unknown music styles. They can quickly adapt to a different style and are able to adjust their expectations after a short exposure to an unknown style. There are currently two different prevailing explanations for such behavior: (1) when listening to music, we continuously learn the characteristics of the style as we pay attention to the stylistic tendencies in music and (2) there are basic psychological principles or universal qualities of music that can be applied to various music styles (Krumhansl et al., 2000; Huron, 2008).

The aforementioned research shows the interconnection between music perception and culture, focusing on comparing the perception of different music features: from “low-level” rhythmic and melodic complexity, to “high-level” features, such as mood and emotion. Through exploration of perception between different cultures, users' familiarity with culture-influenced music style was evaluated and analyzed. However, the users' expertise, which could affect perception, was seldom explored in these studies. In this paper, we explore both the effect of expertise and familiarity, and their influence on human perception of music by observing user responses in a melody prediction task.

### 2.2. Computational Melody Prediction

In music theory and information retrieval, music patterns and their repetitions have been studied by a number of groups, both in theory (e.g., Lerdahl and Jackendoff, 1983; Margulis, 2013) and computational approaches (e.g., Marsden, 2010; Ren, 2016; Pesek et al., 2017b). Several tasks emerged throughout the years in the Music Information Retrieval Evaluation eXchange (MIREX), which is a community-based framework for formal evaluation of algorithms and techniques related to music information retrieval (MIR) (Downie, 2008). Among a variety of available MIREX tasks, the *Discovery of repeated themes & sections* (Collins et al., 2014) and *Patterns for prediction* became popular in the last decade. The aim of the *Discovery of repeated themes & sections* task is to find repetitions, which represent one of the more significant aspects of a music piece (Meredith et al., 2002). The MIREX task definition states “the algorithms take a piece of music as input, and output a list of patterns repeated within that piece” (Collins, 2016). Based on this discovery task, the *Patterns for prediction* task was created as an offshoot of the pattern discovery task (Janssen et al., 2019). The goal of this task is to predict a continuation of a given excerpt. There are two subtasks defined within the *Patterns for prediction* task: in the *explicit task*, the algorithm is given an excerpt and should generate a continuation; in the *implicit task*, the algorithm is given an excerpt and a candidate continuation, and should return the probability that this is the true continuation of the provided prime.

Several authors tacked the *Pattern for prediction* subtasks in recent years. de Reuse (2019) proposed a CopyForward algorithm for the explicit task, inspired by the Cosiatec approach by Meredith (2013). The CopyForward algorithm selects a section of the excerpt, and translates and copies it as a continuation. Colombo (2018) also proposed an approach named BachProp for the explicit task, which uses a recurrent neural network to determine note probabilities for the continuation. Ycart and Benetos (2019) proposed an LSTM model for the implicit task, which discriminates the real and fake continuations, while Ens and Pasquier (2019) proposed the GenDetect algorithm, which generates a collection of categorical distributions for each music excerpt and the uses a Gradient Boosting Classifier, to predict whether a continuation is real or fake.

We proceed by describing two computational models for modeling musical expectations that we used in our study: the Implication-Realization (I-R) model by Narmour (1990) and the Compositional Hierarchical Model for symbolic music representations (SymCHM) by Pesek et al. (2017b). The I-R model represents one of the most well known models of melodic expectation. It is based on musicological rules, so we deem it has high expertise and is culturally agnostic. As an alternative, we chose the SymCHM model, which is based on compositional modeling through unsupervised learning. It thus deduces the underlying rules from the music itself and does not incorporate any direct musicological knowledge in its structure. The two models represent two opposite poles and well-fit our goal of exploring the differences between expertise and familiarity in prediction.

#### 2.2.1. Implication-Realization (I-R) Model

Eugene Narmour studied Leonard Meyer's theory of musical expectation, based on the understanding of musical structure and the perception of musical emotions and meaning. He then developed a complex theory of melodic perception, which he called the Implication-Realization Model (Narmour, 1990).

The model considers *implicative* intervals, by which one forms an expectation about the continuation of the melody, and the *realized* intervals, which (presumably) fulfill these expectations (Toiviainen and Eerola, 2016). The model therefore observes the perceptive systems which process information in a *top-down* manner, as well as those which process information in a *bottom-up* manner. While the latter approach represents the expectations, the former mimics the realization of the representations, which are learned and depend on the musical knowledge and culture of an individual (Pearce, 2003).

The I-R model contains five criteria on the basis of which the suitability of the realized interval is estimated:

- registral direction: implicit intervals larger than 8 semitones imply a change in the direction of the melody, and those smaller imply preservation of the direction,
- registral return: it prefers returns to the first tone of the implicit interval or a deviation from the latter by a maximum of 2 semitones in any direction,
- intervallic difference: Implicit intervals of 5 or more semitones imply similarly large realized intervals (with a deviation of up to 2 semitones in any direction when changing the direction of the melody, or three semitones in the direction of conservation); while the implicit intervals which are >5 semitones imply smaller realized intervals,
- proximity: this criterion prefers the realized intervals of five semitones or smaller,
- closure: implies a change in the direction of the melody or a smaller realized interval than the implicit interval, if the latter was large (at least 3 semitones larger than the former).

The model prefers small realized intervals and preserves the direction of the melody or stays on the same tone, and in the case of larger realized intervals, a change in the direction of the melody.

#### 2.2.2. Updated I-R Model

The five basic criteria of Narmour's model were later updated by additional criteria by different researchers, most notably Schellenberg (1997), who reduced the number of factors. These criteria are not all based on the realization of implicit intervals. The added criteria are:

- consonance: the preferred realized intervals are the consonant intervals: unison, perfect forth, perfect fifth, and octave (Krumhansl, 1995),
- tonality: tonally more stable tones are preferred (Krumhansl, 1999),
- melodic attraction: the tonal ratio of tonality of both tones in the realized interval (Lerdahl, 1996),
- tessitura: predictions of tones that are close to the median pitch of the melody (Hippel, 2000),
- mobility: on the basis of the auto-correlation between consecutive tones, this criterion estimates how predictive the individual tone is in terms of previous tones and the median position (Hippel, 2000).

In the experiment, we used the bottom-up I-R model's implementation provided by Toiviainen and Eerola (2016) to compute the values for the selected criteria.

#### 2.2.3. Compositional Hierarchical Model for Symbolic Music Representations (SymCHM)

In recent years, deep architectures based on neural networks have become prominent in the field of machine learning and pattern recognition. Such architectures have the ability to learn and model characteristics of the underlying data on multiple levels of abstraction, with simple structures being modeled on low levels and more complex concepts on higher layers.

The current implementations of neural network-based deep architectures need large amounts of data for training, and are thus less usable in cases where the amount of data is limited. Moreover, these approaches operate as black boxes, as insight into the learned structures is difficult and the ability to use these models for observation and analysis is therefore limited.

The Compositional Hierarchical Model for music information retrieval was first introduced by Pesek et al. (2014) as a deep architecture that can learn to model the input data on multiple layers with increasing levels of complexity, but also has the ability to learn from small datasets, and enables insight into the learned structures, including automated chord estimation and multiple fundamental frequency estimation (Pesek et al., 2017a). The model was built on a time-frequency-magnitude input and produced frequently co-occurring compositions of harmonic structures in the input signal. The symbolic version of the model SymCHM (Pesek et al., 2017b) has been applied to the task of finding repeated melodic patterns and sections by learning for compositions of symbolic events in the time-pitch-onset domain. The model has also recently been applied to the extraction of rhythmic patterns (Pesek et al., 2020). The SymCHM learns a hierarchical representation of patterns occurring in the input, where patterns encoded by the parts on higher layers are compositions of the patterns on lower layers. “Part activations” expose the learned patterns (and their variations) in the input data. Shorter and more trivial patterns naturally occur more frequently, longer patterns less frequently. On the other hand, longer patterns may entirely subsume shorter patterns.

The motivation for such model originates in the idea of decomposition of complex signals into simpler parts of the signal. These parts possess various levels of granularity and can be distributed across several layers, depending on their complexity. Starting from the first layer, which contains parts representing individual events (such as a frequency presence, or a note event in symbolic representation), new consecutive layers of frequently co-occurring compositions are created. The consecutive layers thus contain compositions of parts on previous layers. Since statistics is employed as the driving force behind the training procedure of such model, the structure can be learned in an unsupervised manner.

The learned hierarchy is also transparent. By observing the learned concepts encoded in individual compositions, the structure can be transparently observed without a specialized process, which is needed in black-box architectures. The learned concepts in the compositional hierarchy are encoded relatively. Again, originating from the idea of signal decomposition, parts with the same structure occur in different signals, as well as at multiple time- and pitch/frequency-shifted locations in a single signal. By relatively encoding the structures, a single composition is activated at multiple locations of the learned concept occurring in the input signal. The activations therefore represent the instantiations of the relatively encoded concepts.

##### 2.2.3.1. Training and using the SymCHM

Starting at its input, the model observes the event co-occurrence frequencies and relatively models the relations between them. In terms of melodic sequences, if two or more events co-occur on a specific interval in several locations, both events can be joined into a composition. The latter represents a newly composed part on a consecutive layer. The composition is relatively encoded, meaning that should two events co-occur at one pitch location and again at a different one, the same composition is formed. This procedure is repeated layer-by-layer until the desired complexity of the learned parts is achieved. In contrast to the first layer where the model observes individual events in the input, co-occurrences of compositions are observed on higher layers. These then form new relatively encoded compositions on a consecutive layer, based on the previous layer.

Each part may occur at several locations in the input. Since the part is relatively encoded, the occurrences are defined by temporal placement and pitch attributes. The occurrence of a part in the input is denoted “activation,” which contains the information about the time and pitch of the occurrence. The parts learned by the model can be observed as melodic patterns and their activations as pattern occurrences.

Once the model is built, it can be inferred over another (or over the original input). The inference may be exact or approximate, where in the latter case biologically-inspired hallucination and inhibition mechanisms enable the model to find variants of part occurrences with deletions, changes, or insertions, thus increasing its predictive power and robustness. The hallucination mechanism provides means to activate a part even when the input is incomplete or changed. In symbolic music representations, such changes often occur in melodic variations and ornamentation. The hallucination enables the model to robustly identify patterns with variations. The inhibition mechanism is also essential in SymCHM for the removal of redundant co-occurrences. As the model does not rely on any musicological rules, parts may produce a large number of competing patterns. Inhibition may be used to reduce the number of activations and find the patterns that best correspond to the learned hierarchy.

SymCHM therefore learns a hierarchical representation of patterns occurring in the input, where patterns encoded by the parts on higher layers are compositions of the patterns on lower layers. The inference produces “part activations” which expose the learned patterns (and their variations) in the input data. Shorter and more trivial patterns naturally occur more frequently, longer patterns less frequently. On the other hand, longer patterns may entirely subsume shorter patterns.

### 2.3. Tonal Hierarchies

The theory of tonal hierarchies by Krumhansl and Cuddy (2010) is based on the assumption that statistically frequent musical patterns (in most cases) provide reliable guidelines for the listener's abstraction of the tone hierarchy. The listeners should therefore successfully orient themselves to the actual tonal hierarchy. Their perception should also coincide with the frequencies of the occurrence of tones and their combinations.

Musical context establishes the tonal hierarchy. Certain tones are more specific, more stable and more important to the structure than others. In classical Western tonal-harmonic music of the eighteenth and nineteenth centuries, tonic is the main tone in the tonal hierarchy, followed by the dominant, dominant parallels, the remaining tones of the scale, and lastly the tones that are not part of the scale. This hierarchy reflects the influence of a triadic (acordic) structure in which consonant chords dominate. Krumhansl and Cuddy (2010) used the *probe tone* method to quantify tone hierarchies. The participants were asked to listen to incomplete scales and evaluated how well the individual tones completed the scale. The results are shown in Table 1.

**Table 1:** The major and minor tonal hierarchies, obtained by the probe tone method.

**Note**	**C**	**C#**	**D**	**D#**	**E**	**F**	**F#**	**G**	**G#**	**A**	**A#**	**B**
C-major	6.35	2.23	3.48	2.33	4.38	4.09	2.52	5.19	2.39	3.66	2.29	2.88
C-minor	6.33	2.68	3.52	5.38	2.60	3.53	2.54	4.78	3.98	2.69	3.34	3.17

Tones higher in the tonal hierarchy appear more often and last longer and in the stressed metric positions (Krumhansl, 1999). Moreover, the higher their position in the hierarchy, the more quickly these tones are recognized as part of the scale (Janata and Reisberg, 1988).

In addition to the musical reference points that lead the musical perception, musical memory and understanding, the listeners are also sensitive to frequently occurring sound sequences (Saffran et al., 1999; Saffran and Griepentrog, 2001). By repeated implicit listening, they develop mental representations that reflect musical consistency, through which they then encrypt and memorize musical patterns, and generate expectations. Sensitivity to these consistencies allows for a relatively quick adaptation to new musical styles. The concept that one central tone is a reference point for a multitude of hierarchically connected tones is not limited to the Western tonic-harmonic style, but also to other styles and cultures. Unique hierarchies can even be found within individual songs.

Western listeners quickly adapted to the tonal hierarchies of an unknown (Indian) style in an experiment performed by Castellano et al. (1984). It turned out that the more important tones were played many times, which allows the listeners who are not familiar with the style to find the appropriate tonal hierarchy. Even the inexperienced listeners are flexible and adapt quickly to tone sequences in unknown musical contexts, while for the musically educated participants, the statistical processing of music becomes even more evident (Oram and Cuddy, 1995).

## 3. Methodology

In this paper, we present two studies on how melody prediction is affected by music familiarity and expertise in (1) human listeners and (2) computational approaches. In this section, we first describe two datasets of music excerpts we collected and used in both studies. We also describe the evaluation metrics used to assess prediction accuracy, and the translation and validation of the Music Sophistication Index, used as an instrument to assess human music expertise. Finally, we describe how we used the SymCHM by adjusting it for the melody prediction task.

### 3.1. Datasets

To control for music familiarity, we collected two datasets of folk songs to be used in our studies: Chinese and European. The Chinese music excerpts (used with permission of the authors) are part of the music database used in the study of the perception of musical phrases by Nan et al. (2006). The European sections were taken from the freely available online collection Robokopp^2^, which contains folk and war songs and anthems from German and English speaking environments. Since we conducted the first study on Slovenian participants, we limited the selection to German songs, due the German influence on Slovenian folk songs and the Slovenian musical heritage (Vodušek, 1959; Vidakovič and Delo, 2003).

#### 3.1.1. Generating the Excerpts

We initially randomly selected 30 non-polyphonic musical fragments in each of the datasets. We converted the MIDI song representations into audio using the Midi Sheet Music and MuseScore 2 programs. We re-synthesized all of the MIDI excerpts in order to avoid variations in sound and timbre quality between both collections.

**Figure 1:**
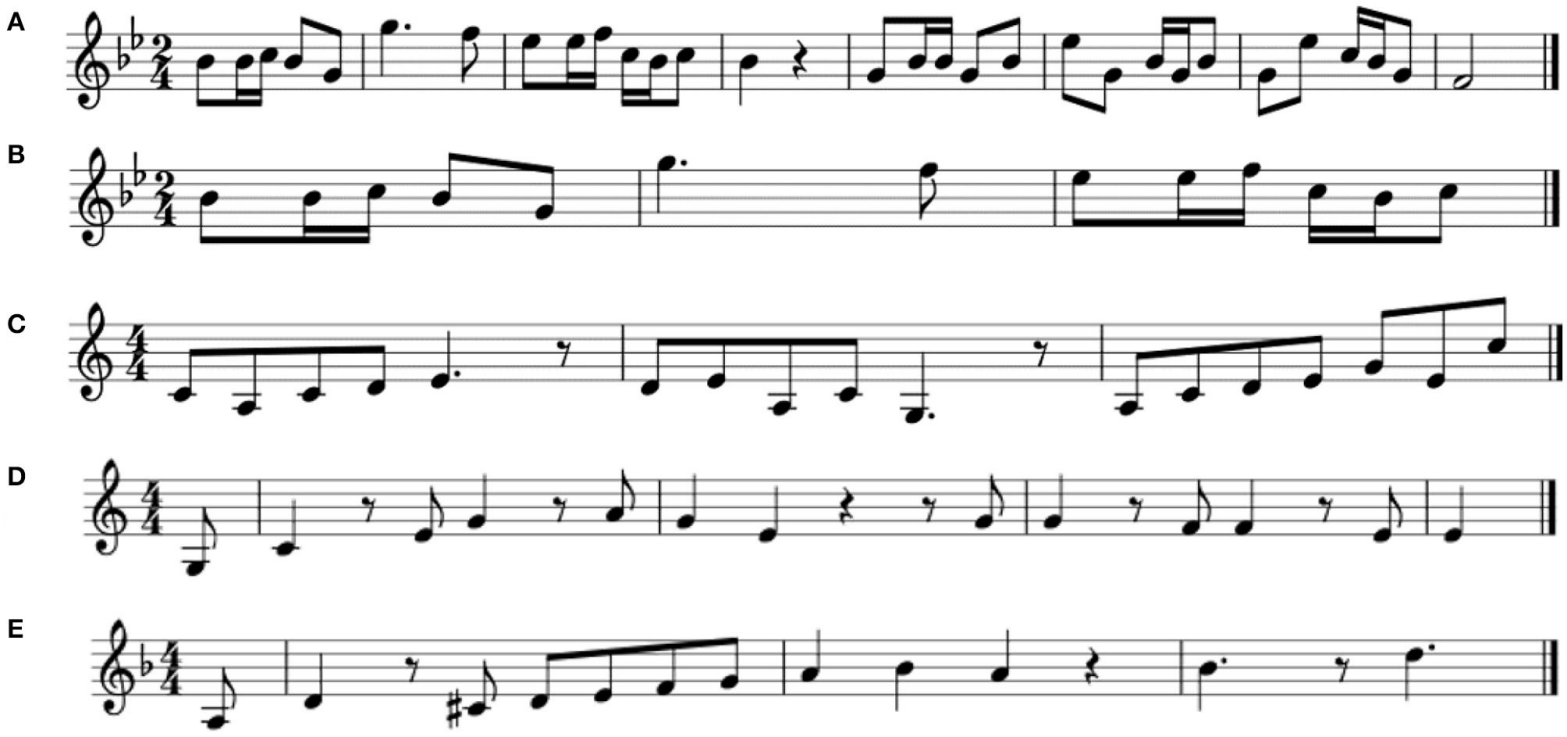
**(A)** A full Chinese folk song; **(B)** A Chinese song excerpt; complete (excerpt 1); **(C)** A Chinese song excerpt, incomplete (excerpt 2); **(D)** A European song excerpt, complete (excerpt 3); **(E)** A European song excerpt, incomplete (excerpt 4).

As the average length of the songs was 18.7 s (about 8 bars), we created shorter song excerpts, representing individual phrases within the songs, to make the music prediction task more user-friendly. An example of a full song is shown in Figure 1A, while its shortened excerpt is depicted in Figure 1B.

We shortened the songs in two different ways: some of the songs were cut after the penultimate tone in the phrase (complete-phrase excerpts—the participants had to predict the last tone in the phrase), while others were cut at random (incomplete-phrase excerpts). The dataset contained 75% of the complete-phrase excerpts and 25% of the incomplete-phrase excerpts.

From the initial 30, we chose 20 excerpts per dataset based on the following criteria: (1) the number of tones in the excerpt, (2) the maximum interval occurring in the excerpt, and (3) the tonal range of the excerpt.

In order to ensure a homogeneous structure of musical events, we selected fragments that were within two standard deviations of each criterion across the dataset. The values for the four excerpts described in Figure 1 are given in Table 2.

**Table 2:** Values of the three criteria for the music excerpts b–e, depicted in Figure 1.

**Excerpt**	**Number of events**	**Largest interval**	**Range in semitones**
b	13	VIII (octave)	12
c	17	VI (sixth)	17
d	15	VI (sixth)	12
e	12	IV (fourth)	11

#### 3.1.2. Analysis of the Generated Datasets

We used one-way ANOVA to compare the chosen criteria of the musical excerpts between the two datasets. The differences between the datasets were not statistically significant (Table 3).

**Table 3:** ANOVA comparison of the German and Chinese datasets.

**Criterion**	***MS***	***F*_**(1, 38)**_**	**p**	**ω^2^**
Number of events	28.9	1.88	0.179	0.021
Largest interval	0.1	0.04	0.845	−0.025
Tonal range	6.4	0.54	0.469	−0.012
Duration in seconds	4.2	1.04	0.315	0.001

*1−β = 0.11 at α = 0.05*.

The musical excerpts contained 15 events on average (Chinese: μ = 15.6, σ = 3.3, German: μ = 13.9, σ = 4.3). The lowest number of events in the Chinese dataset was 10, while the highest was 22. In the German dataset, the lowest number of the events was 7, while the highest was 24.

The largest interval in the majority of songs was the major sixth. Across the Chinese dataset, the largest intervals ranged from a minor third to the octave. On the European dataset, the songs' largest interval ranged from fourth to the tenth (decima). The datasets differed only slightly in the tonal range. The average range of the songs was 12 semitones (Chinese: μ = 12.4, σ = 3.8; German: μ = 11.6, σ = 2.8). In the Chinese dataset, the smallest range was five semitones and the largest range was 20 semitones. In the German dataset, and the smallest range was 7 semitones and the largest 17.

### 3.2. Evaluation Metrics

As mentioned earlier in the problem formulation, a sequence of melodic events can have different continuations that make sense from a music theory perspective. This is demonstrated in different songs that share a part of the melody, which at some point diverge. Hence, a predicted tone, which makes musical sense, but is not the exactly the same as in the original melody, should be considered as correctly predicted.

**Figure 2:**
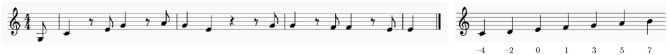
A list of suitable octave-invariant responses for the selected excerpt from the European dataset in C-major.

**Figure 3:**
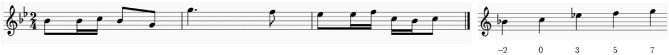
A list of suitable octave-invariant responses for the selected excerpt from the Chinese dataset in the pentatonic scale starting in B♭.

In our experiment, we devised two evaluation metrics: strict evaluation, which considers only the correct note, and relaxed evaluation, where a prediction, which is part of the tonal hierarchy (scale) of the music excerpt, is taken as correct. Thus, for excerpts in the European dataset in a major scale, seven tones are correct (see Figure 2), while for those in a minor scale, nine tones are correct (including the augmented sixth and seventh scale degrees appearing in the harmonic and melodic scales). For the excerpts in the Chinese dataset, the five suitable continuation notes are based on the pentatonic scale (Figure 3).

### 3.3. Slovenian Translation of the Music Sophistication Index

In order to measure the music expertise of the subjects, we used the Goldsmiths Music Sophistication Index (Gold-MSI) instrument (Müllensiefen et al., 2014). The Gold-MSI is a questionnaire with 38 items that measure various aspects of music sophistication. It was developed for English speaking subjects. Because the subjects in our study were Slovenian-speaking and the instrument was not available in Slovenian, we needed to adapt it.

When a questionnaire is adapted to another cultural environment it has to be validated (Sousa and Rojjanasrirat, 2010). The intercultural differences can affect the validity of the participant's responses in a questionnaire in a situation where non-native speakers are asked to respond, even if they possessed good knowledge of the language (Blažica and Lewis, 2015). Moreover, the differences in the participants' responses can occur even when using the same questionnaire in two cultures with a single language, but with completely different cultures (e.g., the USA and New Zealand) (Brown et al., 2017). Therefore, in addition to translation, adaptation and the assessment of validity and reliability (test–retest reliability and internal consistency) are obligatory for the adaptation of an instrument for a new cultural environment (Arafat et al., 2016).

#### 3.3.1. Translation and Implementation

The Gold-MSI questionnaire contains 38 self-report items about musical engagement and education. The first 31 items contain statements and the individuals assess on a 7-point Likert-type scale how much they agree with each of the statements, while the last seven items ask about their music education, the number of instruments played by the individual and similar.

The questionnaire was translated into Slovenian with multiple quality checks, as suggested by Sousa and Rojjanasrirat (2010). Two translators independently translated the questionnaire and the third translator reviewed the translation. We combined both translations into the first Slovenian version. This version was back-translated by the fourth (independent) translator into English. Finally, we compared back-translation with the original English text and implemented minor changes to the first version. The final Slovenian translation was implemented as an online questionnaire, using the PHP framework CodeIgniter. In addition to the Gold-MSI questionnaire, four short demographic questions (gender, age, education, status) were asked. The data were processed using the statistical analysis tool R.

#### 3.3.2. Participants

The questionnaire was completed by 231 people (79 men, 152 women). The participants were mostly students (136) and employees (75), aged between 16 and 58 (μ = 26.7, σ = 7.3). Almost all participants (96.5%) had education higher than secondary school: 83 had undergraduate degree (3-year programme equivalent to the first Bologna cycle), 86 completed the graduate programme (second Bologna cycle), and 54 the postgraduate programme (third bologna cycle).

The vast majority of the participants had at least a few years of music education. Only 66 participants never attended a music school (28.6%). The majority (*f* = 139, 60.2%) were enrolled in some formal type of education for at least three years, of which 44 participants had 10 or more years of music education. One third of the participants (*f* = 75) never learned about music theory, whereas 128 participants (55.4%) were trained in this field for three years or more (of which 41 participants had more than six years of experience). In Slovenian music-school system, the elementary music education most commonly takes 6 years and involves both learning a music instrument and music theory courses. Only 38 participants (16%) had no music experience at all—they never attended a music school, nor did they learn to play any instruments by themselves.

Most of the participants answered that they a attended music school for singing (*f* = 47), followed by piano (*f* = 43), guitar (*f* = 33), flute (*f* = 15), and violin (*f* = 11). The other instruments had a frequency of 3 or less, and 65 participants (28.1%) did not play any instrument.

#### 3.3.3. Confirmatory Factor Analysis

First, we compared the characteristics of the translated and original version of Gold-MSI. We checked the gathered responses in terms of seven parameters—average values, dispersion (minimal and maximal values, standard deviations), Cronbach's α, McDonald's ω, and Guttman's λ6 for five different categories reported by Müllensiefen et al. (2014):

- active engagement—A,
- perceptual abilities—P,
- musical training—M,
- singing abilities—S,
- emotions—E.

Each of the 31 questions in the questionnaire belongs to one of these five categories: A and P contain 9 questions each, M and S contain 7, and E contains 6. We also considered the general sophistication factor (GEN), which includes 18 of the 31 questions.

We cross-examined the values reported in the initial research and compared them to the results we had obtained. The cross-examination is shown in Table 4. The reliability measures indicate good internal consistency in all factors, both for the English and the Slovenian versions.

**Table 4:** Comparison of average values, dispersion, and reliability measures between the research performed by Müllensiefen et al. (2014) (EN; *n* = 147.633) and our research (SL; *n* = 231) for five specific factors and the general factor of musical sophistication.

	**Active engagement (A)**		**Perceptual abilities (P)**		**Musical training (M)**		**Singing abilities (S)**		**Emotions (E)**		**General sophistication (GEN)**	
	**EN**	**SL**	**EN**	**SL**	**EN**	**SL**	**EN**	**SL**	**EN**	**SL**	**EN**	**SL**
*M*	41.52	37.67	50.20	50.85	26.52	28.65	31.67	32.22	34.66	34.77	81.58	82.16
*SD*	10.36	11.69	7.86	9.67	11.44	12.20	8.72	10.18	5.04	5.83	20.62	23.93
Max	63	62	63	63	49	48	49	49	42	42	126	124
Min	9	9	9	22	7	7	7	8	6	14	18	22
α	0.87	0.87	0.87	0.88	0.90	0.92	0.87	0.88	0.79	0.76	0.93	0.94
ω	0.87	0.88	0.87	0.89	0.90	0.92	0.87	0.88	0.79	0.77	0.93	0.94
G6	0.86	0.87	0.87	0.88	0.91	0.93	0.87	0.88	0.77	0.74	0.94	0.96

A one-way *t*-test was used to compare the results of the Slovenian sample on all five specific factors of musical sophistication and the general factor with the average for each factor obtained in the original survey. The values were statistically significant (*p* < 0.05) for the active engagement factor and the musical training factor (Table 5), indicating the presence of the differences between the Slovenian and original sample in these types of musical sophistication, measurement non-invariance (e.g., differences in interpretation of values on the Likert scale) and similar.

**Table 5:** Results of one-way *t*-test for average values of individual factors.

**Factor**	***t*_**(230)**_**	***p***	***d***
Active engagement	−5.01	<0.001	−0.661
Perceptual abilities	1.02	0.309	0.135
Musical training	2.65	0.009	0.349
Singing abilities	0.82	0.415	0.108
Emotions	0.29	0.774	0.038
General sophistication	0.37	0.713	0.049

We also performed a confirmatory analysis of the Slovenian questionnaire, as the authors of the initial research report a poor fit to the one-factor model (Müllensiefen et al., 2014). Confirmatory factor analysis of the one-factor model of the Slovenian questionnaire has shown that the data did not fit well, χ^2^(665)=2901, *p* < 0.001; CFI = 1.00; TLI = 1.00; RMSEA = 0.150, 90% CI = [0.146, 0.154]; SRMR = 0.128. The fit was even worse with the 5-factor model (the same factors were used as in the original survey), χ^2^(655)=9690, *p* < 0.001; CFI = 0.328; TLI = 0.278; RMSEA = 0.244, 90% CI = [0.240, 0.249]; SRMR = 0.085, which was one of the reasons why we had to re-construct the questionnaire.

#### 3.3.4. Exploratory Factor Analysis

The aim of the exploratory analysis was to reduce the number of items that would still give sufficient information about the music sophistication of the participants. Since the experimental task in our research was quite long, we intended to shorten the Gold-MSI questionnaire significantly.

We extracted one general factor with the eigenvalue of 13.1. We then selected the items with absolute factor loading greater than 0.70; the questionnaire was thus reduced to eight items (its length was reduced by 79%).

Based on these eight items, we created a new index of musical sophistication (Table 6), which coincides well with the general sophistication index of Gold-MSI (*r* = 0.95). The correlation was calculated on the basis of the score obtained by adding the weighted values of individual items (the number of points for the individual index of musical sophistication was the sum of the items no. 5, 7, 10, 12, 19, 22, 27, and 32, weighted by their loading on the extracted factor; Gold-MSI was also calculated using the same procedure). The final version of the short Slovenian Gold-MSI contained items on the ability to make judgments about good singing, abilities to play music/sing by heart, sing proper notes, compare two versions of the same song, recognize specifics of the music piece and detect wrong notes, as well as items on identifying with being a musician and the amount of practicing an instrument.

**Table 6:** Selected items with the highest loading.

**No.**	**Item**	**Factor loading**
5	Dobro znam presoditi, ali je nekdo dober ali slab pevec. (*I am able to judge whether someone is a good singer or not*)	0.734
7	Na pamet lahko pojem ali igram skladbe. (*I can sing or play music from memory*)	0.753
10	Ob spremljavi glasbenega posnetka sem sposoben zapeti prave note. (*I am able to hit the right notes when I sing along with a recording*)	0.794
12	Zmožen sem primerjati in razpravljati o razlikah med dvema izvedbama ali različicama iste pesmi. (*I can compare and discuss differences between two performances or versions of the same piece of music*)	0.795
19	Zmožen sem prepoznati posebnosti poslušane skladbe. (*I am able to identify what is special about a given musical piece*)	0.802
22	Opazim, kadar nekdo poje ali igra napačne tone. (*I can tell when people sing or play out of tune*)	0.724
27	Ne bi rekel, da sem glasbenik. (*I would not consider myself a musician*)	0.746
32	Koliko časa ste redno, dnevno vadili glasbeni inštrument? (*I engaged in regular, daily practice of a musical instrument (including voice) for X years*)	

*The first column corresponds to the item number in the original Gold-MSI questionnaire. The second column describes the individual items in Slovenian*.

### 3.4. Adapting the SymCHM for Melody Prediction

The SymCHM model was initially designed for pattern discovery in symbolic music data, so we adapted it for the melody prediction task. In the following subsection we describe the adaptation of the model used in the experiment.

The implementation of SymCHM works with a comma-separated-values (.csv) input, commonly used in the MIREX pattern discovery task. We therefore transformed the MIDI files from the dataset to the desired CSV files as follows. A song is represented by a single CSV file. Each line in the CSV file contains the following three elements {*T*_*o*_, *P*_1_, *D*}, where individual variables represent the following features:

- *T*_*o*_: onset time
- *P*_1_: pitch
- *D*: duration time.

As SymCHM performs pattern matching, we needed to convert the discovered patterns, represented by the part structures in SymCHM, into melody predictions. We thus perform prediction by searching for occurrences of the patterns, which the model learned during training. We search for individual learned patterns in a given excerpt, and find the continuation of the melody from the best fitting patterns.

To perform this search, we converted the found patterns into regular expressions of relatively encoded patterns with gaps. These gaps represent differences between the learned pattern and the identified pattern. For example, a relatively encoded pattern {0, 5, −3, 0} was transformed into the following regular expression: → {0[0*a* − *zA* − *Z*]*E[0*a* − *zA* − *Z*]*c[0*a* − *zA* − *Z*]*0}. The pattern represented a melodic structure in which the second event occurs five semitones above the first, third event occurs three semitones below the first event, and the fourth event occurs at the same position as the first event. The positive semitone offsets were encoded into upper-case letters (e.g., 5 → *E*), and the negative offsets into lower-case letters (e.g., −3 → *c*). The [0*a*−*zA*−*Z*]* segments (i.e., sequences of notes) represented gaps of indefinite length. These gaps allowed the discovered patterns to match with potential variations in the patterns.

For each excerpt, we searched for the learned patterns of the SymCHM model, encoded as regular expressions. By excluding the last event and matching all the remaining events in the pattern, we were able to obtain the “predicted” pitch. The procedure is shown in Table 7, where the midi pitch represents the MIDI sequence of melodic events. Since there were a number of gaps in the regular expressions, we assigned weights to the predictions using the following criteria, increasing their prediction probability:

- the length of a pattern,
- the proximity of the beginning of the pattern toward the end of the excerpt,
- the total length of matched gaps.

**Table 7:** The procedure of pattern matching and weight calculation.

**Position**	**16**	**15**	**14**	**13**	**12**	**11**	**10**	**9**	**8**	**7**	**6**	**5**	**4**	**3**	**2**	**1**
MIDI Pitch	64	62	64	62	64	62	71	71	71	72	71	69	74	74	72	?
Segment	0	b	0	b	0	b	G	G	G	H	G	E	J	J	H	*
Pattern 1	0		0		0											**D**
Segment							0	0	0	A	0	b	C	C	A	*
Pattern 2							0	0	0		0	b	C	C		**A**
Segment												0	E	E	C	*
Pattern 3												0	E	E		**L**

*Letters represent relatively encoded pitch values*.

Using the criteria, the following formula was established to calculate the weight (relevance) of the individual predictions

(1) $$\begin{array}{r} w=\frac{N_{eventsinpattern}}{N_{segmentlength}-N_{beginningofthematch}+1} \end{array}$$

For a pattern with 32 events which would completely match the last 32 events in the segment, the calculated weight is $w=\frac{32}{33}=0.97$. For a 4-event pattern, matching the last 4 events in a segment, the weight would be smaller—$w=\frac{4}{5}=0.60$). A 4-event pattern which would match the first event in the 20-event segment, the weight is considerably lower—$w=\frac{4}{21}=0.19$.

Pattern 1 in Table 7 contains an example with a low weight: $w_{1}=\frac{4}{17}=0.24$. None of the shown patterns matched completely, with patterns 2 and 3 matching better than pattern 1—$w_{2}=\frac{8}{11}=0.73$ and $w_{3}=\frac{4}{6}=0.67$.

## 4. Studies

In this section, we present the two studies, where we assess how music familiarity and music expertise influence melody prediction in human listeners, and, expressed in appropriate data/algorithmic ways, computational models. The datasets of music excerpts presented in the Methodology section were used in both studies. The Slovenian translation of the Music Sophistication index was used in Study 1. In both studies we used both the strict and relaxed evaluation measures.

### 4.1. Study 1: Human Melody Prediction

In this study, we researched the influence of the participants' expertise on their predictions. We first collected their responses and, based on their music sophistication, split them into two groups of musicians and non-musicians. We explore the differences in their prediction through several types of prediction evaluation. Based on their self-report on familiarity with Chinese music, we also compare their responses to assess the impact of familiarity.

#### 4.1.1. Data Acquisition

To acquire the data, we created a web interface that consisted of two parts: in the first part we gathered demographic information, musical expertise (using the shortened Gold-MSI questionnaire), music preferences and the frequency of listening to Chinese music. In the second part we asked the participants to perform the melody prediction task.

During the first part the participants were asked about their age, gender and level of education, followed by eight questions from the short Gold-MSI questionnaire, as described in Table 6. The participants were also asked to choose up to three preferred genres from a list of 20 genre labels. Additionally, they reported on the amount of time (daily, a few times a month, a few times a year, never) they listened to Chinese music.

**Figure 4:**
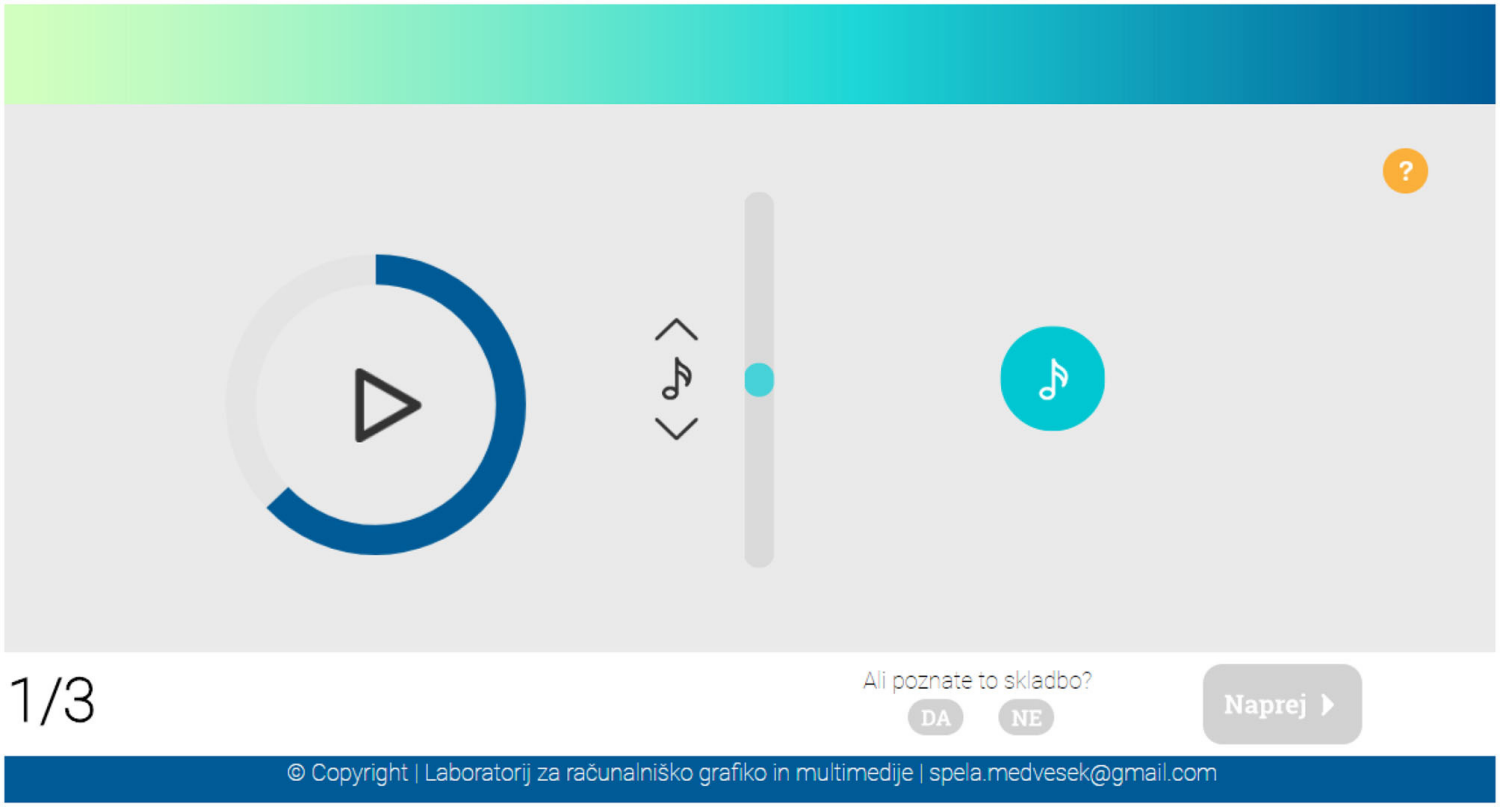
The user interface developed for the prediction part of the questionnaire. When the play button was depressed, the excerpt was played. By dragging the vertical scroll-bar, a pitch in between ±12 semitones was played. By clicking on the note button on the right side, the chosen pitch was added to the end of the excerpt. When added, the excerpt with the added pitch could be played back by clicking on the play button on the left.

In the second part, the participants' task was to predict the continuation of melodic sequences from the database using an interface shown in Figure 4. The task of the participants was to listen to a short music excerpt, and then select the pitch they believed best continued the excerpt. The participants could choose a pitch between ± 12 semitones of the last pitch in the excerpt. If necessary, the participants could listen to the excerpt and their selected continuation combined. There were no time limit or number of replays imposed on the participants. In the end, they had to indicate whether or not they had recognized the song. By asking this question, we wanted to avoid the noise induced by responses of participants who knew the songs in the dataset. In case of known songs, the participants would most likely correctly predict the continuation of the excerpt. In this experiment, we evaluated how shared melodic patterns of European folk songs contribute to the familiarity, and avoid known prediction of known songs.

The participants first learned how to use the interface with three trial music excerpts. We added this step to give the participants the time to familiarize themselves with the interface, in order to minimize the influence of the interface on the responses. The chosen songs were a well-known children's song (*Kuža pazi*), the national anthem (*Zdravljica*) and the international version of “Happy Birthday.” Because these songs are well known in their environment, the participants were able to focus on the interface during the trial usage.

After the initial interaction, the participants proceeded to the experimental part, during which they listened to the excerpts from the Chinese and the German song datasets. The order of the datasets and the excerpts within was randomized to exclude the bias of the dataset order. The questionnaire was distributed via email and social networks.

#### 4.1.2. Participants

Fifty-seven participants, 26 male and 31 female, completed the questionnaire. The participants were between 16 and 54 years old (μ = 26.7, σ = 7.5). The majority of the participants (59.6%) had the education level higher than a high-school diploma, of which 14 obtained a bachelor's degree, 16 had a master's degree and 10 had a PhD degree. All participants were Slovenian.

A large majority of the participants frequently practiced a musical instrument in the past. Only 17.5% never practiced an instrument. The average practice span was 6.8 years (σ = 6.5). A third of the participants (33.3%) practiced for more than 10 years (the maximum was 25 years), and about three quarters (73.7%) practiced at least for three years. The most popular music genres chosen by the participants were classical music—13 participants chose this genre as their favorite, 5 participants chose it as second favorite and 15 as their third favorite genre. Other most popular genres were rock (in order of top three favorite genres: 16, 6, and 4), and pop (6, 9, 5).

The participants mostly did not listen to Chinese music: 39 participants (68%) never and 13 rarely listened to it. Only two participants listened to Chinese music regularly (a few times a month) and three participants listened to it almost daily.

#### 4.1.3. Participant Groups

To distinguish between familiarity and expertise, we split the participants into two groups. The first group contained participants with high music sophistication (we refer to them as *musicians*), based on the music sophistication index and their performance in the trial attempts of the melody prediction questionnaire. The participants in the second group (the *non-musicians*) scored lower on the MSI questionnaire (below 39 points, the average MSI score was 42.2 out of 56) or missed the prediction for either of the three commonly-known songs in the trial attempts of the second part of the questionnaire. There were 36 participants assigned into the first group (17 male, 19 female, MSI: μ = 46.8, σ = 3.3; years of music education: μ = 9.6, σ = 6.3), while the second group contained 21 participants (9 male, 12 female, MSI: μ = 34.4, σ = 6.8; years of music education: μ = 1.8, σ = 2.2).

#### 4.1.4. Influence of Expertise on Predictions

On average, the participants correctly predicted 58% of European and 34% of Chinese continuations. For the European excerpts, they were statistically significantly better (*V* = 135, *p* < 0.01) at predicting the complete sections (Table 8, *European complete excerpts*) in comparison to the incomplete sections, while for the Chinese dataset they were better at solving the incomplete excerpts, but the difference was not significant (*V* = 571, *p* = 0.523).

**Table 8:** The performance of the participants and the computational models in melody prediction task, using strict evaluation.

	**European excerpts**			**Chinese excerpts**		
	**All (%)**	**Complete (%)**	**Incomplete (%)**	**All (%)**	**Complete (%)**	**Incomplete (%)**
All participants	58	63	42	34	34	35
Musicians	68	74	47	39	37	44
Non-musicians	41	43	33	25	27	21
baseline *(all notes)*	4	4	4	4	4	4
baseline *(scale notes)*	6.7	6.7	6.7	9.1	9.1	9.1
SymCHM–eu	60	73	20	30	33	20
SymCHM–cn	45	53	20	30	40	0
Adjusted I-R model	50	60	20	35	25	40

*The table also includes a baseline, taking into account all possible 25 semitons, which could be picked from the interface, and a scale notes baseline, which includes 15 semitones for major/minor scales (European excerpts), and 11 semitones for pentatonic scale (Chinese excerpts), both within ± one octave*.

The musicians performed much better than the non-musicians, for both the European (68 vs. 41%) and the Chinese dataset (39 vs. 25%). The differences between both groups were statistically significant for music of both datasets and both excerpt types. The greatest difference between musicians and non-musicians occurred between the prediction of the European complete excerpts (Wilcoxon test—European dataset: complete excerpts, *W* = 613.5, *p* < 0.01; incomplete *W* = 536.5, *p* = 0.01) and the Chinese incomplete excerpts (Wilcoxon test—Chinese dataset: complete excerpts, *W* = 537.5, *p* = 0.01; incomplete excerpts, *W* = 609.5, *p* < 0.01).

#### 4.1.5. Relaxed Evaluation

We also analyzed the responses through relaxed evaluation. The participants were better at correctly predicting both the European and the Chinese excerpts, with 94.5% success for European dataset, and 89.7% for Chinese dataset. For the subgroups on the European dataset, the results were in favor of musicians (97.9%) vs. non-musicians (88.6%); both groups were slightly more successful when predicting the continuations of the complete musical excerpts (98.3 and 89.5% for the musicians and non-musicians, respectively) than the incomplete ones (96.7 and 85.7%). On the Chinese dataset the musicians achieved 95.6% (with 95.7% of the complete excerpts and 95.0% of the incomplete excerpts continued correctly according to the relaxed evaluation), while the non-musicians achieved 79.7% (80.9% for complete and 76.2% success rate for incomplete excerpts).

#### 4.1.6. Assessing Familiarity With Music of Foreign Culture

One of the questions on the demographic part of the questionnaire was how often the participants listened to Chinese music. Three responded that they listen to it practically every day, two responded a few times a month, and 13 occasionally (a few times a year). Therefore, 18 participants reported that they listen (at least occasionally) to Chinese music, and 39 reported that they never listen to it.

To analyze the differences between familiarity and expertise, we split the participants into two subgroups, depending on their self-report about the familiarity with Chinese music. Considering their sophistication, there was a similar ratio (2:1) of musicians vs. non-musicians in both groups (Table 9).

**Table 9:** Comparing the performance of the participants who listen to Chinese music to those who do not listen to Chinese music.

	**Musicians**			**Non-musicians**		
**Listens to Chinese**	**All (%)**	**Complete (%)**	**Incomplete (%)**	**All (%)**	**Complete (%)**	**Incomplete (%)**
Yes (12 musicians, 6 non-musicians)	38	37	40	24	26	20
No (24 musicians, 15 non-musicians)	40	37	49	25	26	21

Differences between the musicians and the non-musicians of Chinese music were not statistically significant. This indifference can imply that we chose a prevalent culture with a very specific musical style, which the participants have heard before at least a few times (for example, in Chinese restaurants, popular movies, etc.), and that, with minimum exposure, the participants were able to memorize the characteristics of the style well-enough to perform this task.

### 4.2. Study 2: Computational Melody Prediction

In this subsection, we analyze the performance of the two selected computational models: the SymCHM and the I-R model. We also compare their melody prediction performance with the participants' results from study 1.

The SymCHM model needs to be trained before it can be used for prediction, and we decided to train two different models, each on a separate folk song dataset: one only on European folk songs (SymCHM–eu) and one only on Chinese folk songs (SymCHM–cn). In this way, we can estimate how (un)familiarity with a certain culture influences the model. The training sets contained approximately 14,000 events (tones) from the Essen folk song collection^3^ and did not contain the songs from the generated excerpts used for the melody prediction task. In the study, we additionally evaluate the influence of the training set's size on prediction, thus controlling for the different amounts of “familiarity” with a music culture.

The SymCHM model extracts all knowledge from its training set. In contrast, the I-R model needs no training, as it contains universal music-theoretical rules, derived from human expertise. The I-R model therefore represents an expert system without any culture-specific familiarity.

#### 4.2.1. Comparison of Expertise and Familiarity in Computational Models

##### 4.2.1.1. SymCHM

The SymCHM, trained on the set of European songs (SymCHM–eu), correctly predicted 60% of the European excerpts and 30% of the Chinese excerpts. It also correctly predicted 73% of complete excerpts in the European dataset and 20% of the incomplete excerpts. For the Chinese dataset, the distribution was more uniform: the SymCHM correctly predicted 33% of the complete and 20% of the incomplete excerpts.

The SymCHM trained on the set of Chinese songs (SymCHM–cn), was less successful than the SymCHM–eu. It is interesting that SymCHM–cn performed better on the European dataset than the Chinese dataset, correctly predicting 45% of the European excerpts and, the same as SymCHM–eu, only 30% of the Chinese excerpts.

Considering the complete and incomplete excerpts, the SymCHM–eu performed better on the complete excerpts: 73% complete and only 20% incomplete excerpts were correctly predicted on the European dataset; and 33 % complete and 20% incomplete excerpts were correctly predicted on the Chinese dataset.

##### 4.2.1.2. Influence of the Learning Dataset Size on SymCHM's Results

All the reported results of the SymCHM–eu and SymCHM–cn models were obtained using models trained on approximately 15,000 events (300 short European songs for SymCHM–eu, and 189 longer Chinese songs for SymCHM–cn). Considering the learned familiarity, we evaluated the SymCHM model's performance using different smaller dataset sizes. Initially, we trained the SymCHM–eu model with only 50 songs. To assess the model's performance with respect to the dataset size, we trained the model using larger datasets, thus increasing its musical knowledge. The results are shown in Table 10.

**Table 10:** The impact of the SymCHM–eu's training dataset size, repeated 5 times (for training sizes 50–200).

**No. of songs**	**50**	**100**	**200**	300*
Avg. no. of events	2k	4.5k	9.5k	15k
Avg. – all excerpts	37% σ = 6.8	39% σ = 11.0	42% σ = 9.7	53% σ = 9.4
Avg. – complete excerpts	45% σ = 7.8	45% σ = 4.9	55% σ = 10.9	64% σ = 12.3
Avg. – incomplete excerpts	12% σ = 9.8	4% σ = 10.0	8% σ = 9.8	20% σ = 0

The training was repeated only 3 times for the training set of 300 songs (marked with *).

By increasing the training dataset from 50 to 100 songs, SymCHM's performance was not significantly improved. Moreover, the incomplete sequences were less likely predicted correctly. With the further enlargement of the training set, the model's performance increases faster in the “all” and “complete excerpts,” while it the “incomplete excerpts” increased with the 300-song training set. The performance of the SymCHM is therefore impacted by the training set. The model's performance does gradually increase for all subtypes of exerpts. However, due to the small size of the incomplete excerpts subset, the results vary between training set.

#### 4.2.2. Narmour's Implication-Realization Model

We first evaluated the I-R model. The model's predictions showed, the rules of the initial (non-extended) I-R model are too restrictive and, consequently resulted in poor performance on the melody prediction task. The model it did not correctly predict any continuation on the European dataset, and only three in the Chinese dataset.

We analyzed the ground-truth continuations of the excerpts in the European dataset. These can be summarized three rules regarding the predicted events:

- they are high on the tonal hierarchy,
- if they are on the top or the bottom limit of the excerpt's range, they change direction,
- the distance between the predicted events and the starting point is 7 or less semitones.

These rules are also included in the extended Implication-Realization Model. The initial model listed the 0 (repeating the last event) as the best answer, which is in most cases an acceptable answer from the point of view of conformity with the tonic hierarchy in the song, but it is mostly incorrect. In addition, the I-R model puts too much weight to the answers in the immediate vicinity of the starting point (± a few semitones).

For the purposes of the task, we fine-tuned the model, using a subset of the criteria from the extended Implication-Realization Model, since the initial model emphasized very small intervals and preferred predicting the same tone too often. We retained the *registral return, proximity, tonality, melodic attraction*, and *tessitura* criteria. We obtained the score of each possible outcome by averaging the normalized values of the five criteria.

Using this adjustment of the extended Implication-Realization model, the evaluation yielded significantly better results. The adjusted Implication-Realization Model correctly predicted 50% of European and 35% of Chinese excerpts (Table 8).

#### 4.2.3. Relaxed Evaluation

Taking into account the correct responses in relaxed evaluation, both SymCHM–eu and SymCHM–cn models correctly classified all excerpts in the European dataset. The models therefore achieved better results than the participants. For the Chinese dataset, SymCHM–cn also achieved 100%, while SymCHM–eu achieved only 80%.

#### 4.2.4. Comparison Between the SymCHM and the Adjusted I-R Model

The adjusted I-R model, which was implemented for this experiment using aforementioned libraries, returns a probability score for each of the possible continuations across five different criteria: registral return, proximity, tonality, melodic attraction, and tessitura. All criteria are equally represented and a combined probability is provided for each continuation, given the normalized sum of probabilities across all five criteria. The SymCHM's responses can also be ranked using the weights of the individual responses. We therefore compared both models by observing the ranks that the true continuations receive, with regard to all possibilities. We used the mean reciprocal rank to compare both models.

The mean reciprocal rank (MRR) is a statistical measure for evaluating the accuracy of the order of elements in a list sorted by a criterion (in our case, this is the weight for each possible answer for each song), using the following formula:

(2) $$\begin{array}{r} MRR=\frac{1}{\left|Q\right|}\overset{\left|Q\right|}{\underset{i=1}{\sum }}\frac{1}{rank_{i}}, \end{array}$$

where *Q* represents the list of all possible responses, and *rank*_*i*_ represents the position of the first relevant response. Given the rank of the correct response is 1 (i.e., the first response is the relevant response), its reciprocal rank (RR) equals 1. If the rank of the relevant response is *n*, the RR equals $\frac{1}{n}$.

We calculated the average values for the RR across all three models SymCHM–eu, SymCHM–cn and the I-R model, for the European and the Chinese datasets individually. The results are shown in Table 11.

**Table 11:** Average values for the mean reciprocal ranks (MRR) for all three models on each of the two datasets.

	**Dataset**	
**Model**	**European**	**Chinese**
SymCHM–eu	0.720	0.491
SymCHM–cn	0.635	0.520
Adjusted I-R model	0.648	0.490

The SymCHM–eu obtained the highest MRR, meaning that among the selected models, it was the most successful in providing the responses. For the European dataset, it received an average rank of 0.72, while for the Chinese dataset it received the rank of 0.491. The adjusted I-R model received the RR of 0.648 on the European dataset and 0.49 on the Chinese dataset.

The performance of the adjusted I-R model was more similar across both datasets, compared to the SymCHM's results. Since it contains rules, which are culture agnostic, this behavior was somewhat expected. Nevertheless, the results on the European dataset were still higher. However, the best-performing SymCHM–cn's results were also lower on the Chinese dataset. The performance of the adjusted I-R model and the SymCHM–cn was therefore more consistent across both datasets, compared to the SymCHM–eu model.

## 5. Discussion

During this research, two datasets were collected, each containing 20 music excerpts. The European dataset contained excerpts from the European folk song collection, while the Chinese dataset contained Chinese folk songs. Both the Chinese and the European datasets contained 75% of complete excerpts, in which the final note in the melody or phrase was predicted, and 25% of incomplete excerpts, where the phrase ended at a random location and the following note was to be predicted.

**In study 1**, we first analyzed the collected participants' melody predictions. We further split them in two groups: musicians and non-musicians, based on their MSI score, to analyze the differences between the participants. The MSI questionnaire was translated to Slovenian language and validated (section 3.3).

On average, the European participants correctly predicted 58% of European and 34% of Chinese continuations. For the European excerpts, they were statistically significantly better (*V* = 135, *p* < 0.01) at predicting the complete (Table 8) than the incomplete sections, while for the Chinese dataset they were better at solving the incomplete excerpts but the difference was not significant (*V* = 571, *p* = 0.523).

The participants in the musician group performed much better than the non-musicians, for both the European and the Chinese dataset. The differences between the groups of musicians and non-musicians were statistically significant in the music of both datasets and both complete and incomplete excerpt types. The greatest difference between the musicians and non-musicians occurred between the prediction of the European complete excerpts and the Chinese incomplete excerpts (Table 8). The results confirm the MSI as a credible instrument for music sophistication validation. Since the end of a phrase within an excerpt is more predictable due to the musicological rules of the melodic form, we hypothesized the participants, as well as the models would be more successful in correctly predicting the complete than the incomplete excerpts. The results concur with our hypothesis.

In comparing the responses between the European and Chinese datasets, there were statistically important differences in both groups of participants (musicians and non-musicians). There were also statistical differences in the participants' performance between the musicians and non-musicians within the Chinese, but not European, dataset. The results of significance tests between different groups are shown in Table 12. The better performance of musicians in both datasets was expected in this experiment. We attribute the significance of the difference on the Chinese dataset to the musicians' expertise, whereas the familiarity of the European dataset influenced the relatively better performance of the non-musicians on the European dataset. In this aspect, the comparison of the non-musicians performance on both datasets also unveiled the underlying difference between the listeners' expertise vs. familiarity.

**Table 12:** Results of the Wilcoxon signed-rank test (comparing European and Chinese datasets; W) and Wilcoxon rank sum test (comparing musicians and non-musicians; V).

**Participants**	**Dataset**	***W***	***V***	***p***
Musicians	European : Chinese		0	<0.01
Non-musicians	European : Chinese		32	<0.01
Musicians : non-musicians	European	370		0.90
Musicians : non-musicians	Chinese	152		<0.01

Regardless of the poorer performance of the participants in both groups on the Chinese dataset (compared to the European dataset), their performance was quite high—the participants predicted a suitable response in almost 90% of cases, while the musicians achieved an almost perfect score.

**In study 2**, we performed an evaluation of the prediction of the two computational models, and compared them to the participants' prediction performance. In general, the participants performed better in the prediction task than the compared models on both datasets. It is evident that SymCHM, especially SymCHM–eu, came very close to the performance of the participants. We can conclude that the SymCHM model's performance lays between non-musicians' and musicians' performance in this prediction task. The extended I-R model first needed fine-tuning to perform in this task. After the adjustment, the model performed significantly better. However, it seems the expertise implemented in this model does not outperform the participants, nor the SymCHM, which was trained and thus familiar with the background.

In the relaxed evaluation of the predictions, both the SymCHM–eu and SymCHM–cn models correctly classified all excerpts in the European dataset, and therefore achieved better results than the participants. This can be attributed to the learning process in which the SymCHM extracts the common patterns from the training dataset. If the dataset only contains a major/minor or pentatonic scales, the model will output only predictions matching the scales. In this aspect, these results should not be generalized. In a similar manner, the relative simplicity of the pentatonic scale (compared to the major/minor scales) influenced the SymCHM–cn model's results. The SymCHM–cn achieved a 100% success in the relaxed evaluation on the European dataset and the SymCHM–eu achieved lower results on the Chinese dataset. We attribute these results to in the structure of the scales—the tones of the pentatonic scale are a subset of the diatonic scale. In the relaxed evaluation, both the adjusted I-R model and the SymCHM model performed worse on the Chinese dataset than on the European dataset. We did not expect this difference in performance, since the SymCHM model learns the patterns from the training set, while the adjusted I-R model employs rules, which are universal to music. In this sense, we could attribute this difference to potential Western-music bias, although further research is needed to confirm this assumption.

We also compared the predictions using the mean reciprocal rank. On the European dataset, the SymCHM–eu performed significantly better than the adjusted I-R model (second best) and the SymCHM–cn. On the contrary, the SymCHM–cn received the best score on the Chinese dataset, whereas the SymCHM–eu and the adjusted I-R model performed similarly worse. The results for the difference between the SymCHM–eu and the SymCHM–cn models were expected: the models were trained, and therefore familiarized, with each music type, and were therefore expected to perform better when applied to the music of the same cultural background. On the other hand, the adjusted I-R model performed similarly on both datasets, proving the I-R model is agnostic of music culture. Therefore, the difference between the experience and the familiarity in computational approaches is clearly visible in their performance across different datasets.

Additionally, the SymCHM models were also evaluated by training on different dataset sizes. It is evident, that the model performs better, when trained on a larger dataset, thus increasing its “familiarity” with the underlying patterns, which are shared among the songs with similar cultural background.

The role of familiarity due to cultural background has been discussed in related work. Although some works support the assumption that cultural background plays a role in experiencing music (Balkwill and Thompson, 1999; Cross, 2001; Morrison et al., 2003; Demorest et al., 2008; Soley and Hannon, 2010) recent work has shown that within-culture variance of music is higher than between-culture variance (Mehr et al., 2019). This would indicate that the specific choice of music from a culture may influence how much variance the familiarity variable accounts for.

## 6. Conclusion

In this paper, we explored the influence of the listeners' cultural background and their music sophistication on melody prediction. This was done on two datasets consisting of musical excerpts of European and Chinese folk songs. The melody prediction data was gathered on 57 participants. The participants were asked to predict the possible melody continuation of each music excerpt from the two datasets. The responses were split into two groups: (i) musicians (high music sophistication), and (ii) non-musicians (low music sophistication). The music sophistication was acquired using the MSI instrument, which was adapted to Slovenian-speaking participants. We compared the participants' responses of the two groups. Musicians performed better than non-musicians on both the familiar (European) dataset, and the less familiar (Chinese) dataset.

In addition, we compared the melody prediction performance of two computational models: (i) the adjusted I-R model and (ii) the symbolic compositional hierarchical model (SymCHM). The SymCHM was trained twice, once for each melody prediction task (on a set of Chinese songs and European songs, separately). The SymCHM outperformed the adjusted I-R model in the strict melody prediction task. We also compared the predictions of the SymCHM and the adjusted I-R models with the melody prediction performances of human listeners. Musicians outperformed both the SymCHM and the adjusted I-R model.

In both studies, the experiment results showed that the music excerpts which ended at the end of a phrase (complete excerpts) were more predictable than those which ended in the middle of a phrase (incomplete excerpts). Both the participants and the computational models correctly predicted less than half of the incomplete excerpts; they were both more successful in predicting complete excerpts. As the product of this research, we also developed the Slovenian version of the Musical sophistication index questionnaire, evaluated on 230 participants. Additionally, we collected the responses of 57 participants in the prediction task. Both the dataset and the translated MSI questionnaire are made publicly available.

Based on the described work, we are planning on extending this experiment with participants from different cultural backgrounds and with datasets of less-known folk music. We also plan on performing an inverted experiment to further assess the computational models, by using the models' predicted responses and having the participants evaluating their subjective correctness of the responses. Another planned extension of the current research will be exploring the participants' latent factors which influence their implicit expertise in predicting. Furthermore, we plan on further exploring the error patterns of human listeners and evaluate the underlying decision process in comparison to their music sophistication.

## Data Availability Statement

The original contributions presented in the study are included in the article/supplementary materials, further inquiries can be directed to the corresponding author/s.

## Ethics Statement

Ethical review and approval was not required for the study on human participants in accordance with the local legislation and institutional requirements. The patients/participants provided their written informed consent to participate in this study.

## Author Contributions

MP and MM wrote a plan for research. ŠM executed experiments. AP examined the results in Study 1. MP, MT, and MM evaluated the results in Study 1. MP, MT, and ŠM wrote the article. All authors contributed to the article and approved the submitted version.

## Conflict of Interest

The authors declare that the research was conducted in the absence of any commercial or financial relationships that could be construed as a potential conflict of interest.

## Footnotes

^1^The reader may find a lot of similar sets of songs, for example The Kinks: All Day And All Of The Night vs. The Doors: Hello, I Love You or the triple Mike Oldfield: Tubular Bells vs. Death Angel: The Ultra-Violence vs. Possessed: The Exorcist

^2^http://www.musicanet.org/robokopp/Volksong.html (accessed February 15, 2018)

^3^http://kern.ccarh.org/browse (accessed June 22, 2018)
